# Admixture Mapping of African–American Women in the AMBER Consortium Identifies New Loci for Breast Cancer and Estrogen-Receptor Subtypes

**DOI:** 10.3389/fgene.2016.00170

**Published:** 2016-09-21

**Authors:** Edward A. Ruiz-Narváez, Lara Sucheston-Campbell, Jeannette T. Bensen, Song Yao, Stephen Haddad, Christopher A. Haiman, Elisa V. Bandera, Esther M. John, Leslie Bernstein, Jennifer J. Hu, Regina G. Ziegler, Sandra L. Deming, Andrew F. Olshan, Christine B. Ambrosone, Julie R. Palmer, Kathryn L. Lunetta

**Affiliations:** ^1^Slone Epidemiology Center, Boston University, BostonMA, USA; ^2^College of Pharmacy, The Ohio State University, ColumbusOH, USA; ^3^College of Veterinary Medicine, The Ohio State University, ColumbusOH, USA; ^4^Department of Epidemiology, Gillings School of Global Public Health, University of North Carolina at Chapel Hill, Chapel HillNC, USA; ^5^Department of Cancer Prevention and Control, Roswell Park Cancer Institute, BuffaloNY, USA; ^6^Department of Preventive Medicine, Keck School of Medicine, University of Southern California/Norris Comprehensive Cancer Center, Los AngelesCA, USA; ^7^Rutgers Cancer Institute of New Jersey, New BrunswickNJ, USA; ^8^Cancer Prevention Institute of California, FremontCA, USA; ^9^Division of Cancer Etiology, Department of Population Science, Beckman Research Institute, City of Hope, DuarteCA, USA; ^10^Sylvester Comprehensive Cancer Center and Department of Public Health Sciences, University of Miami Miller School of Medicine, MiamiFL, USA; ^11^Epidemiology and Biostatistics Program, Division of Cancer Epidemiology and Genetics, National Cancer Institute, BethesdaMD, USA; ^12^Vanderbilt Epidemiology Center, Vanderbilt University and the Vanderbilt-Ingram Cancer Center, NashvilleTN, USA; ^13^Department of Biostatistics, Boston University School of Public Health, BostonMA, USA

**Keywords:** breast cancer, African American women, admixture mapping, genetics, fine-mapping

## Abstract

Recent genetic admixture coupled with striking differences in incidence of estrogen receptor (ER) breast cancer subtypes, as well as severity, between women of African and European ancestry, provides an excellent rationale for performing admixture mapping in African American women with breast cancer risk. We performed the largest breast cancer admixture mapping study with in African American women to identify novel genomic regions associated with the disease. We conducted a genome-wide admixture scan using 2,624 autosomal ancestry informative markers (AIMs) in 3,629 breast cancer cases (including 1,968 ER-positive, 1093 ER-negative, and 601 triple-negative) and 4,658 controls from the African American Breast Cancer Epidemiology and Risk (AMBER) Consortium, a collaborative study of four large geographically different epidemiological studies of breast cancer in African American women. We used an independent case-control study to test for SNP association in regions with genome-wide significant admixture signals. We found two novel genome-wide significant regions of excess African ancestry, 4p16.1 and 17q25.1, associated with ER-positive breast cancer. Two regions known to harbor breast cancer variants, 10q26 and 11q13, were also identified with excess of African ancestry. Fine-mapping of the identified genome-wide significant regions suggests the presence of significant genetic associations with ER-positive breast cancer in 4p16.1 and 11q13. In summary, we identified three novel genomic regions associated with breast cancer risk by ER status, suggesting that additional previously unidentified variants may contribute to the racial differences in breast cancer risk in the African American population.

## Introduction

A majority of the close to 100 breast cancer risk loci that have been identified to date were found using genome-wide approaches in populations of European ancestry women (Maxwell and Nathanson, 2013; Michailidou et al., 2015; Couch et al., 2016). Follow-up studies in non-European populations are necessary to understand the population-specific risk associated with these loci. Further, genome-wide associations studies in non-European populations are required to identify race-specific genetic variation associated with breast cancer subtypes (Feng et al., 2014). Sample size remains one of the main limiting factors in performing genome-wide association studies (GWAS) in non-Europeans. Admixture mapping, a method for discovery of genomic regions that is based on linkage disequilibrium in populations with recent population admixture, requires far fewer samples and markers than GWAS to detect loci associated with phenotypic variation (Rosenberg et al., 2003; Smith and O’Brien, 2005; Winkler et al., 2010). Early admixture mapping efforts in African American women with breast cancer were likely under-powered, in particular to detect loci associated with breast cancer subtypes (Fejerman et al., 2009). However, this approach, in conjunction with fine mapping, has been successful at identifying genetic associations with other complex traits in recently admixed populations (Zhu et al., 2005; Freedman et al., 2006; Nalls et al., 2008; Manuck et al., 2011; Bensen et al., 2014; Jeff et al., 2014; Parker et al., 2014).

African American women have higher breast cancer mortality rates than US women of European ancestry, particularly among younger women, attributable to diagnosis at a later stage often with larger tumors and lymph node metastases (Chlebowski et al., 2005; Hershman et al., 2005; Grann et al., 2006; Fejerman and Ziv, 2008). In addition, women of African ancestry are at greater risk of having estrogen receptor negative (ER-) and triple negative (ER-, progesterone receptor negative, PR-; and human epidermal growth factor receptor 2 negative, HER2-) breast cancer at all ages compared with European American women (DeSantis et al., 2014). ER- and triple negative cancers are more aggressive, and have fewer treatment options than ER+ disease (Clarke et al., 2012). These differences in incidence, severity and mortality between women of African and European ancestry suggest that there may be different underlying genetic contributions to breast cancer risk in African American and European American women. Recent genetic admixture coupled with these striking racial differences provides excellent rationale for performing admixture mapping in African American women with breast cancer.

To this end, we performed a genome-wide admixture mapping in germline DNA in the African American Breast Cancer Epidemiology and Risk (AMBER) Consortium, a collaborative project from four of the largest studies of breast cancer in African American women (Palmer et al., 2014a). This represents the largest breast cancer admixture mapping study in African American women to date. We discuss our findings in the context of those from the previous admixture mapping study of breast cancer (Fejerman et al., 2009) and of known breast cancer risk loci identified among European Americans recently confirmed in African Americans.

## Materials and Methods

### Ethics Approval and Consent to Participate

The CBCS was approved by the Institutional Review Board at the University of North Carolina at Chapel Hill School of Medicine. The WCHS was approved by the Institutional Review Boards at the University of Medicine and Dentistry of New Jersey (presently Rutgers University), Mount Sinai School of Medicine, and Roswell Park Cancer Institute. The BWHS was approved by the Institutional Review Board at the Boston University School of Medicine. The MEC was approved by the Institutional Review Boards of the University of Hawaii and University of Southern California. Written informed consent was obtained from each participant.

### Study Population

Two case-control studies, the Carolina Breast Cancer Study (CBCS) and the Women’s Circle of Health Study (WCHS), and two prospective cohort studies, the Black Women’s Health Study (BWHS) and the Multi-Ethnic Cohort (MEC) comprise the AMBER Consortium (Palmer et al., 2014a,b; Bandera et al., 2015); the four studies are described below.

The CBCS is a population-based case control study of women ages 20–74, conducted in 44 counties in North Carolina that began in 1993 (Newman et al., 1995). Breast cancer cases were identified using Rapid Case Ascertainment in cooperation with the NC Central Cancer Registry. Controls were identified using Division of Motor Vehicles lists (for women under age 65) and Health Care Financing Administration lists (for women 65 and older). The age range of study participants was 20–74 years. CBCS contributed 1,408 African–American cases and 615 controls to this analysis.

The WCHS includes both European and African American women, 20–75 years of age, newly diagnosed with a first primary, and histologically confirmed breast cancer. Cases were identified from 10 counties in New Jersey through the New Jersey State Cancer Registry and through major metropolitan hospitals in New York City serving a large minority population. Controls, frequency matched to cases by 5-years age categories and self-reported race, were identified using random digit dialing in New York City and New Jersey, as well as from the community (Ambrosone et al., 2009; Bandera et al., 2013). Recruitment in New York City took place between 2003 and 2008. Recruitment at New Jersey started in 2006 and is ongoing. WCHS contributed 821 African American breast cancer cases and 834 African American controls to this analysis.

The BWHS is an ongoing prospective cohort study of 59,000 US women aged 21–69 years from across the United States who self-identify as “black” (Rosenberg et al., 1995). Beginning in 1995, women completed a 14-page postal health questionnaire. Biennial postal questionnaires are mailed to participants to update information. During 2004–2007, we obtained saliva samples as a source of DNA from 26,814 participants using the mouthwash-swish method (Cozier et al., 2004). BWHS contributed 901 cases and 2,249 controls to this analysis.

The MEC study is a prospective cohort that includes over 215,000 individuals from Hawaii and California (primarily Los Angeles) assembled between 1993 and 1996 (Kolonel et al., 2000; Pike et al., 2002). African Americans, Native Hawaiians, Japanese, Latinos and European Americans comprise the predominant population. Blood samples were collected from incident breast cancer cases starting in 1994 and identified by cohort linkage to Surveillance, Epidemiology and End Results (SEER) registries, as well as a random sample of MEC participants to serve as controls for genetic analyses. MEC contributed genetic data from 499 African American cases and 960 African American controls to this analysis.

### Genotyping and Quality Control

Genotyping of constitutional DNA of CBCS, WCHS, and BWHS samples was performed by the Center for Inherited Disease Research (CIDR) using the Illumina Human Exome Beadchip v1.1 array with custom content. The standard content of this array includes more than 240,000 coding variants, as well as tag SNPs for GWAS hits, a grid of common variants, and ancestry informative markers (AIMs). A more complete description of the exome chip design and standard content is available from http://genome.sph.umich.edu/wiki/Exome_Chip_Design.

Genotype quality control for this study has been described previously (Laurie et al., 2010). Genotype data were available for 2,624 autosomal AIMs (**Supplementary Table 1**) included as standard content in the exome array and typed in AMBER participants, including 3,130 cases, 1,674 ER+ cases and 963 ER- cases (515 triple negative) and 3,698 controls. In addition, genetic data from 499 cases (130 ER- including 86 triple negative, 294 ER+, 75 ER unknown) and 960 controls in the MEC study were available from previous genotyping on an earlier version of the exome array that contained the same AIMs. Final sample size for the current admixture analyses was 8,287 participants: 3,629 cases (1,093 ER- including 601 triple negative, 1,968 ER+, and 568 ER status unknown) and 4,658 controls.

We assessed population stratification by calculating principal components of genetic variation using the smartpca program in the EIGENSOFT package (Patterson et al., 2006). Supplementary Figure 1 shows a plot of the top two principal components of the participants stratified by study. No obvious differences in distribution of these principal components were observed across studies.

### Admixture Mapping

We used ADMIXMAP software version 3.8.3103 (Hoggart et al., 2004; Montana and Pritchard, 2004; Reich and Patterson, 2005) to estimate African locus-specific ancestry and determine excess sharing across the genome. ADMIXMAP uses a Markov chain Monte Carlo algorithm to generate a score test at each position in the genome that measures local ancestry associations with a phenotype (Hoggart et al., 2004). Admixture mapping is generally carried out using case-only and/or case-control analyses. The case-only approach compares locus-specific ancestry in each chromosomal position with average genome-wide ancestry. The case-control method tests for differences of locus-specific ancestry between cases and controls adjusting for individual admixture and other covariates. Case-only analysis has greater statistical power than case-control assuming that deviation in ancestry between cases and controls is not due to population stratification (Shriner, 2013). We therefore first performed a case-only admixture scan and then a case-control admixture analysis to confirm local ancestry associations from the case-only results. Case-control analyses were adjusted for global individual admixture, study, age (10-year groups), geographic region, and source of constitutional DNA (Oragene-saliva, blood, mouthwash-saliva). Statistical significance was assessed using *Z* statistics, with a threshold of |*Z*| > 4.0 considered genome-wide statistically significant. A negative *Z-*score indicates that higher African (lower European) ancestry at that particular locus is associated with higher breast cancer risk while a positive *Z-*score indicates that lower African (higher European) ancestry is associated with higher breast cancer risk. We performed analyses of breast cancer overall, and by ER and triple negative status. For each analysis, we ran ADMIXMAP using 2,000 burn-in iterations and 8,000 follow-on iterations. The percentage of African ancestry for each individual was determined using ADMIXMAP and compared across controls, all cases and by ER status as well as by site, using logistic regression adjusting for the covariates described above.

### Association Analyses

In order to identify SNPs that may help explain any observed association between local African ancestry and risk of breast cancer regions meeting the genome-wide significant criterion (|*Z*| > 4) in AMBER, fine-mapping analyses were performed using typed and imputed data from the African American Breast Cancer (AABC) consortium, which included participants from six epidemiological studies (Chen et al., 2011, 2013) after excluding MEC, WCHS, and CBCS subjects which were included in AMBER. Regional association analyses were conducted in 2,114 cases (1,116 ER+, 591 ER-, and 407 unknown ER status) and 1,911 controls typed on the Illumina Human1M-Duo BeadChip [34] and imputed using MACH with HAPMAP2 as a reference. Within each genome-wide significant admixture peak a Bonferroni correction for the number of directly genotyped markers tested was used to determine statistical significance (Palmer et al., 2016).

## Results

**Table 1** shows the participant characteristics by study site in cases and controls. A total of 3,629 breast cancer cases (1,968 ER+ cases, 1,093 ER- cases including 601 triple negative, and 568 unknown ER status) and 4,658 controls were included in the present analysis. CBCS and WCHS cases had the highest proportion of African ancestry (84.0%) and MEC the lowest (78.0%). Mean African ancestry, adjusted for age, geographic region, DNA source and study site was 81.3% for controls and 81.2% for cases (*P* = 0.74 for difference of means). A total of 2,624 autosomal AIMs were analyzed (**Supplementary Table 1**).

**Table 1:** Characteristics of participants by study in the AMBER consortium.

Characteristic	BWHS		CBCS		WCHS		MEC		Total	
	Cases	Controls	Cases	Controls	Cases	Controls	Cases	Controls	Cases	Controls
Total	901	2249	1408	615	821	834	499	960	3629	4658
% African ancestry^a^	80.5	81.4	84.0	83.5	84.0	84.0	78.0	78.1	81.2	81.3
% Family history^b^ of breast cancer	15	9	18	11	16	12	21	13	17	11
ER status										
ER+	498		741		435		294		1968	
ER-	233		565		165		130		1093	
Triple negative	102		380		33		86		601	
Unknown	170		102		221		75		568	
Age at diagnosis										
<40	47		204		85		0		336	
40–49	262		459		215		9		945	
50–59	302		381		292		108		1083	
60–69	204		267		173		165		809	
≥70	86		97		56		217		456	

*ER, estrogen receptor; BWHS, Black Women’s Health Study, CBCS, Carolina Breast Cancer Study, WCHS, Women’s Circle of Health Study, MEC, Multi-Ethnic Cohort. ^a^Mean of individual African ancestry (%) adjusted for age, geographic region, DNA source, and study site. ^b^First-degree family history of breast cancer.*

### Admixture Mapping

**Table 2** shows the four genome-wide significant regions in case-only analysis (|*Z*| ≥ 4, *P* < 6 × 10^-5^) that also had confirmatory results in case-control analysis (|*Z*|≥ 2.0, *P* < 0.05). We identified two novel genome-wide significant regions, showing evidence of excess African ancestry associated with ER+ breast cancer on 4p16.3 and 17q25.1. The 10q26 and 11q13.2 regions both contain SNPs previously identified in published GWAS of breast cancer in European ancestry women (Hunter et al., 2007; Turnbull et al., 2010), and fine-mapping efforts have identified independent signals in African American women (Chen et al., 2011).

**Table 2:** Genome wide significant regions of excess African ancestry (negative *Z*-score) and reduced (positive *Z-*score) associated with breast cancer at |*Z*| > 4 in case-only analyses and corresponding case-control results^a^.

Locus	All breast cancers		ER+ breast cancers		ER- breast cancers		TNBC		Breast cancer GWAS SNPs
	*Zc*/*Zcc*	OR^b^ (95% CI)	*Zc*/*Zcc*	OR^b^ (95% CI)	*Zc*/*Zcc*	OR^b^ (95% CI)	*Zc*/*Zcc*	OR^b^ (95% CI)	
4p16.3	-2.4/-0.9	1.05 (0.93–1.18)	-4.1/-2.7	1.22 (1.06–1.41)	0.7/1.6	0.86 (0.72–1.02)	0.7/1.2	0.86 (0.68–1.09)	–
10q26	-4.7/-2.1	1.12 (1.00–1.26)	-3.3/-1.2	1.08 (0.94–1.24)	-3.5/-1.7	1.16 (0.97–1.38)	-1.5/-0.06	0.99 (0.79–1.26)	rs11199914 rs3750817 rs35012336 rs10736303 rs2981579 rs2981578 rs2981575 rs1219648 rs1219642 rs2912774 rs2936870 rs2420946 rs2981582 rs2935717
11q13.2	3.9/1.9	0.91 (0.82–1.00)	4.8/3.1	0.83 (0.75–0.93)	0.5/-0.6	1.04 (0.90–1.21)	-1.1/-1.5	1.16 (0.95–1.42)	rs614367 rs3903072 rs537626
17q25.1	-3.8/-1.7	1.09 (0.98–1.22)	-4.3/-2.6	1.20 (1.05–1.37)	-0.2/0.7	0.93 (0.79–1.11)	-0.6/-0.1	1.00 (0.80–1.26)	–

*^a^|*Z*|≥ 4.0 case-only analysis results are presented only for regions with case-control results |*Z*|≥ 2.0, *P* < 0.05. ^b^Odds ratio and 95% confidence interval per African allele estimated from the admixture mapping case-control analysis. Odd ratios were adjusted for individual African ancestry, study site, age, geographic region, and DNA source. TNBC, triple negative breast cancer. *Zc, Z*-score from the case-only analysis. *Zcc, Z*-score from the case-control analysis.*

**Supplementary Table 2** contains admixture results for all 2,624 AIMs and breast cancer subtypes. **Supplementary Table 3** shows admixture results with suggestive evidence of association, defined as 4 > |*Z*|≥ 3.5 in case-only and |*Z*|≥ 2.0 in case-control analyses. Of these 13 suggestive regions, seven contain at least one genome-wide significant marker previously associated with breast cancer (Hindorff et al., 2015).

### Association Analyses

We used an independent study (African American Breast Cancer Consortium, AABC) to test for association of SNPs in the admixture associated regions identified for ER+ and overall breast cancer. We defined significant association at *P* < 5.8 × 10^-6^ after Bonferroni correction for multiple testing based on adjustment for the 8,653 typed markers included. AABC does not have information about triple negative breast cancer, thus we did not follow up the three regions showing suggestive association with this subtype. We report on the most significant associations in each genomic region identified in the admixture mapping (**Table 3**; Supplementary Figure 2).

**Table 3:** Most significant single independent SNP associations in the African American Breast Cancer (AABC) consortium within genomic regions identified in admixture mapping of AMBER.

Locus/Breast Cancer subtype	Local African ancestry	Position (bp)	Alleles^a^	OR (95% CI)	*P*-value	Effect allele frequency		
						AABC	AFR^b^	EUR^c^
4p16, ER+	Excess				
rs112545418		2279036	A/G	1.46 (1.24–1.72)	4.0 × 10^-6^	0.13	0.14	0.01
10q26, all breast cancer	Excess				
rs12244041		133275151	T/A	1.34 (1.21–1.46)	3.9 × 10^-6^	0.78	0.83	0.68
11q13, ER+	Deficit							
rs77274510		61995513	A/G	3.13 (2.60–3.58)	5.8 × 10^-6^	0.012	< 0.01	0.059
rs117564384		62011320	A/G	2.97 (2.50–3.44)	6.8 × 10^-6^	0.012	< 0.01	0.051
rs116638271		67441627	G/A	2.31 (1.95–2.67)	4.7 × 10^-6^	0.97	0.97	1.0
17q25, ER+	Excess				
rs55850050		74453990	T/C	1.55 (1.23–1.94)	1.6 × 10^-4^	0.062	0.027	0.16

*^a^Effect allele/reference ^b^African ancestry populations from 1000 Genomes Project ^c^European ancestry populations from 1000 Genomes Project*

#### 4p16 and ER-Positive (ER+) Breast Cancer

One SNP in the 4p16 region exceeded multiple test correction threshold, rs112545418 in *ZFYVE28*; the A allele showed a 46% risk increase compared to the G allele (*P* = 4.0 × 10^-6^). Consistent with our admixture mapping results (i.e., excess of local African ancestry at 4p16 associated with higher risk of ER+ breast cancer), the risk A-allele is more frequent in 1000 Genomes African ancestry samples (AFR), 14%, and AABC, 13%, than in European ancestry (EUR) samples, 1% (**Table 3**).

#### 10q26 and Overall Breast Cancer

The most significant association with overall breast cancer, rs12244041, at *P* = 2.9 × 10^-6^, is located over 10 Mb from the well-known breast cancer associated *FGFR2* gene and is unlinked to SNPs in this region (*r^2^* = 0.01). The risk T-allele at this SNP is associated with 87% higher risk of breast cancer, and it is more frequent in AABC (79%) and AFR populations (83%) compared to EUR populations (68%) (**Table 3**). The frequency differences by race are consistent with our admixture mapping results that excess of local African ancestry at 10q26 is associated with higher risk of all breast cancer.

#### 11q13 and ER+ Breast Cancer

Three variants, rs116638271 in *ALDH3B2*, rs77274510 *in RP11-703H8.9*, and rs117564384 in *SCGB1D2* in the chromosome 11q13 region were significantly associated with ER+ breast cancer after correction for multiple testing (**Table 3**). The latter two are only 15,807 bp apart and are in modest LD (*r*^2^ = 0.66) in 1000 Genomes AFR populations, while rs116638271, resides ∼5.4 Mb from rs117564384 and is not correlated (*r^2^* = 0) with either SNP. In agreement with the admixture mapping results (i.e., deficit of local African ancestry at 11q13 associated with higher risk of ER+ breast cancer) the risk alleles of the three SNPs were less frequent in 1000 Genomes AFR vs. EUR populations (**Table 3**). For rs77274510 and rs117564384, the risk alleles had <1% frequency in 1000 Genomes AFR subjects compared to 5.9 and 5.1%, respectively, in EUR samples. For rs116638271 the risk allele had 97% frequency in AFR vs. 100% in EUR. In other words, the protective rs116638271 allele against ER+ breast cancer seems to be specific of AFR populations with a frequency of about 3% but 0% in EUR populations.

The genome wide significant ER+ associated region on chromosome 11 was also suggestive of association in the admixture analyses of overall breast cancer (*Z* = 3.9 in case-only analysis, and *Z* = 1.9 with *P* = 0.058 in case-control analysis). This region contains three overall breast cancer associated variants within an 8.6 Kb region, rs75313515 (*P* = 2.4 × 10^-6^), rs75771004 (*P* = 3.2 × 10^-6^) and rs116667969 (*P* = 3.5 × 10^-6^) that are significant after correction for multiple testing. The first two SNPs are in strong LD while rs116667969 is very weakly correlated with both SNPs (*r*^2^ ∼0.40). The minor alleles range from 2.5 to 4% in our analyses; however, none of the variant alleles at these three SNPs are present in the EUR 1000G populations. These three SNPs are within 10 Kb of rs116638271 (*r*^2^ = 0.40), one of the three SNPs under this peak shown to be associated with ER+ breast, which suggests that this region contains multiple independently associated variants.

#### 17q25 and ER+ Breast Cancer

No single variant was significantly associated with ER+ breast cancer after correction for multiple testing. The risk T-allele, at rs55850050, the most significant SNP in the chromosome 17 region, conferred a 55% increased susceptibility to ER+ breast cancer (*P* = 1.6 × 10^-4^). The direction of the risk was contrary to what was expected based on the admixture results (i.e., excess of local African ancestry at 17q25 associated with higher risk of ER+ breast cancer), as the risk T-allele was less frequent in 1000 Genomes AFR (2.7%) vs. EUR populations (16.1%) (**Table 3**). Notably it resides adjacent to *BRCA1* (17q21.1) and 13 Mb from the 17q23.1 region which contains breast cancer susceptibility SNPs (Ahmed et al., 2009; Kelemen et al., 2009; Lin et al., 2012).

### Regions of Suggestive Genome-Wide Significance

#### Overall Breast Cancer

The 9q34 region showed excess local African ancestry associated with risk of overall breast cancer (**Supplementary Table 3**). It does not harbor known susceptibility breast cancer variants and no SNPs were associated with overall breast cancer at nominal *P* < 0.05. This region contains multiple Crohn’s disease, ulcerative colitis and Type 2 diabetes loci (**Supplementary Table 4**).

#### ER+ Breast Cancer

We found an excess of local African ancestry in the chromosome region 8q24 associated with ER-positive breast cancer (**Supplementary Table 3**). The 8q24 locus contains breast cancer susceptibility loci as well as loci for other cancers including ovarian, prostate, colorectal, glioma, lymphomas and leukemias, bladder and kidney (**Supplementary Table 4**). No single SNP reached significance after correction for multiple testing. Two SNPs, rs147582253 (*P* = 1.9 × 10^-5^) and rs10112657 (*P* = 5.8 × 10^-5^) showed suggestive associations with ER+ breast cancer. The direction of the associations were consistent with the admixture mapping results, as the risk alleles of both SNPs, rs147582253-G (1.7% in African vs. 0% in European populations) and rs10112657-T (3.3% in African vs. 0% in European populations), were more frequent in African ancestry vs. European ancestry populations.

#### ER-Negative (ER-) Breast Cancer

We found three regions, 2q24, 5p13, and 18q23, showing suggestive evidence of association with ER- breast cancer (**Supplementary Table 3**); both 2q24 and 5p13 contain or are adjacent to breast cancer susceptibility variants. The 2q24 region showed a deficit of local African ancestry associated with ER- breast cancer. The admixture signal in 2q24 is less than 4 Mb from two recently identified SNPs, rs2016394, and rs1550623, associated with breast cancer in European ancestry women in the Collaborative Oncological Gene-Environment Study (COGS) (Michailidou et al., 2013). However, these two variants are not specific to ER- breast cancer (Michailidou et al., 2013); thus, they do not explain the admixture signal in 2q24. Our fine-mapping efforts found two independent signals associated with ER- breast cancer. Both protective alleles, rs78550178-G (99% frequency in African vs. 84% in European populations) and rs62172609-G (76% frequency in African vs. 31% in European populations), were more frequent in African ancestry vs. European ancestry populations as expected according to the direction of the admixture signal.

Region 5p15.33, showing excess African ancestry associations with ER- breast cancer, contains the *TERT* gene, which harbors a well-known ER- specific breast cancer associated variant, rs10069690 (Haiman et al., 2011; Garcia-Closas et al., 2013; Michailidou et al., 2013; Purrington et al., 2014). The T allele of rs10069690 was our most significant association with ER- breast cancer (*P* = 2.2 × 10^-5^). The T allele is far more frequent in African populations (66%) versus Europeans (28%). We found some evidence of one additional independent signal in *TERT*, represented by the risk increasing C allele of rs37004 (*P* = 6.8 × 10^-5^, 99% frequency in AFR vs. 78% in EUR) at ∼1 Mb from rs10069690. This variant has been shown to be associated with lung cancer risk in the Genetic Association and Mechanisms in Oncology (GAME-ON) consortium (Karami et al., 2015).

The 18q23 region is novel and showed an excess of local African ancestry associated with risk of ER- breast cancer, however the most significantly associated single SNP, rs58839649 at *P* = 1.5 × 10^-4^, did not pass multiple test correction; the minor G-allele was associated with a 60% increased risk of ER- breast cancer with a frequency in AABC of 10, 13% in AFR populations, and absent in EUR populations. This region contains prostate susceptibility SNPs (Eeles et al., 2013) and has been identified by linkage analyses as a region co-segregating with familial Glioma (Liu et al., 2012) (**Supplementary Table 4**).

#### Triple-Negative Breast Cancer

We found evidence of association of excess African ancestry with triple negative disease at three locations: 2q37.1, 3p24.2, and 18p11 (**Supplementary Table 3**). While higher African ancestry in the 2q37 and 3p24 loci seems to increase risk of ER- breast cancer as well, higher African ancestry in the 18p11 locus appears to be associated only with risk of triple negative breast cancer. The 3p24.2 region contains well-established breast cancer associated SNPs (Matsumoto et al., 1997, 2000; Yang et al., 2001; Ahmed et al., 2009; Lin et al., 2012). However, there are no reports of variants at 2q37.1 or 18p11 associated with breast cancer or breast cancer subtypes. Given these regions of excess African ancestry are associated with disease; it may be possible that these locations harbor breast cancer susceptibility alleles only present in African ancestry populations.

## Discussion

In this large genome-wide admixture scan in African American women, we found evidence of associations of local African ancestry with breast cancer in several genomic regions. Tests of genetic associations in the identified regions in an independent sample suggest that continental ancestry specific genetic variation within these regions may be associated with breast cancer subtypes.

### 4p16 and ER-Positive Breast Cancer

This region contains no previously reported genome wide significant breast cancer loci, but loci for bladder cancer, as well as other traits and diseases include triglycerides and cholesterol levels, bone mineral density, type 2 diabetes and Parkinson’s disease (**Supplementary Table 4**). The most strongly associated SNP in 4p16, rs112545418, in *ZFYVE28*, was in moderate linkage disequilibrium (*r^2^* = 0.48) with a nearby variant rs17132398, associated with ER-positive breast cancer at *P* = 1.8 × 10^-4^. Analysis of rs17132398 using RegulomeDB database (Boyle et al., 2012) showed that this SNP is located inside an enhancer region across multiple cell lines, and is likely to affect binding of transcription factors and expression of *ZFYVE28*, which is a negative regulator of epidermal growth factor receptor (EGFR) signaling (Mosesson et al., 2009). The low frequency of the rs112545418 risk A-allele (1%) and the corresponding risk T-allele of rs17132398 (0.4%) in European ancestry populations may explain why it has not been identified in previous GWAS of breast cancer.

### 10q26 and Overall Breast Cancer

The 10q26 showed excess of local African ancestry associated with risk of overall breast cancer and was our strongest admixture association with overall breast cancer. This region contains well established SNPs in *FGFR2* associated with increased risk of breast cancer (Chen et al., 2011). A recent fine-mapping of *FGFR2* (Meyer et al., 2013) identified three independent signals in Europeans and East Asians: the first signal represented by rs35054928, the second signal by rs45631563, and the third one by rs2981578 (Meyer et al., 2013). The rs2981578 was previously identified as the SNP with the strongest association with overall and ER+ breast cancer in *FGFR2* in African Americans (Chen et al., 2011), and it was also shown recently by us using AMBER consortium data (Ruiz-Narvaez et al., 2016). Interestingly, the excess of African ancestry in breast cancer cases extends over both *FGFR2* and a second region spanning from 131 to 134 Mb on chromosome 10. This second region contains our significant variant, rs12244041 (**Table 3**; Supplementary Figure 2), as well as a non-synonymous variant, rs148966337, previously shown to be associated with both overall and ER- breast cancer (Haiman et al., 2013). This is a less common variant (1.9%) that was only present in the African American women from the MEC study, but completely absent in women of other continental ancestries (Haiman et al., 2013).

### 11q13 and ER-Positive Breast Cancer

Although the chromosome 11q13 region has been previously shown to contain breast cancer SNPs (Michailidou et al., 2013; Ahsan et al., 2014), all three of the SNPs we found within the admixture-identified region were independent of the published GWAS-identified SNPs. One of the three SNPs in the chromosome 11 region, rs117564384, resides in *SCGB1D2. SCGB1D2* expression is high in and almost exclusive to mammary tissue (Culleton et al., 2007), significantly differs between histologically normal breast tissue and breast cancer tissue (Zubor et al., 2015), and it also has been used as a marker to detect disseminated tumor cells (DTC) in breast cancer (Brown et al., 2006; Lacroix, 2006).

In summary, this is the largest admixture analysis of African American breast cancer, both in number of subjects and markers, to date. One limitation of this report is the lack of characterization of tumors by grade and stage that may show differential association with locus-specific African ancestry. Nevertheless, the current study includes more than twice the number cases and a higher density of AIMs throughout the genome compared to the previous admixture mapping of breast cancer in AA women (Fejerman et al., 2009). In addition, the use of controls in the present study allowed us to confirm significant results from the case-only scan. Fine-mapping of the identified regions showed that multiple genomic variants may be within admixture associated regions. Our results suggest that several loci with small to moderate effects rather than a few with strong effects are responsible for the differences of incidence of ER subtypes among US white and black women.

## Author Contributions

ER-N and LS-C performed the statistical analyses and drafted the manuscript. KL supervised the statistical analyses. JB, SY, SH, CH, EB, AO, CA, JP, and KL participated in data analysis and interpretation and provided critical feedback. CH, EJ, LB, JH, RZ, and SD provided data for fine-mapping. CA and JP conceived of the study and participated in the design and coordination of the study. All authors read and approved the final manuscript.

## Conflict of Interest Statement

The authors declare that the research was conducted in the absence of any commercial or financial relationships that could be construed as a potential conflict of interest.

The reviewer JP and handling Editor declared their shared affiliation, and the handling Editor states that the process nevertheless met the standards of a fair and objective review.

## Supplementary Material

TABLE S1 **Number of ancestral informative markers (AIMs) per chromosome**.

TABLE S2 **Admixture mapping results for all 2624 ancestral informative markers and breast cancer subtypes in the AMBER consortium**.

TABLE S3 **Genomic regions with suggestive excess (negative *Z*-Score) and reduced (positive *Z*-score) African ancestry associated with breast cancer at 4 >|*Z*| ≤ 3.5 in case-only analyses and corresponding case-control results**.

TABLE S4 **Phenotypic traits with genome-wide significant associations at the identified breast cancer loci**.
