# Supplementary material for: Admixture Mapping of African–American Women in the AMBER Consortium Identifies New Loci for Breast Cancer and Estrogen-Receptor Subtypes
Source: Front Genet. 2016 Sep 21;7:170. doi: 10.3389/fgene.2016.00170 (PMC5030764; doi:10.3389/fgene.2016.00170)
Supplement: TABLE S1 — Number of ancestral informative markers (AIMs) per chromosome. [file Table_1.DOCX]

| Supplemental Table 1. Number of ancestral informative markers (AIMs) per chromosome | | | |
| --- | --- | --- | --- |
| Chromosome | Chromosome length^a^ (cM) | Number of AIMs | Mean distance^b^ (cM) |
| 1 | 298.7 | 216 | 1.4 |
| 2 | 274.1 | 247 | 1.1 |
| 3 | 244.3 | 193 | 1.3 |
| 4 | 232.0 | 185 | 1.3 |
| 5 | 216.0 | 175 | 1.2 |
| 6 | 217.3 | 159 | 1.4 |
| 7 | 195.0 | 151 | 1.3 |
| 8 | 182.4 | 141 | 1.3 |
| 9 | 195.5 | 116 | 1.7 |
| 10 | 179.8 | 120 | 1.5 |
| 11 | 182.2 | 128 | 1.4 |
| 12 | 182.2 | 125 | 1.5 |
| 13 | 138.2 | 108 | 1.3 |
| 14 | 130.2 | 90 | 1.4 |
| 15 | 140.1 | 76 | 1.8 |
| 16 | 130.7 | 76 | 1.7 |
| 17 | 159.2 | 72 | 2.2 |
| 18 | 129.5 | 69 | 1.9 |
| 19 | 115.8 | 52 | 2.2 |
| 20 | 101.6 | 59 | 1.7 |
| 21 | 66.7 | 32 | 2.1 |
| 22 | 84.5 | 34 | 2.5 |
| Total | 3796.1 | 2624 | 1.4 |
| cM = centimorgans  ^a^ Genetic distance between the first and last AIM of each chromosome  ^b^ Mean genetic distance between consecutive AIMs | | | |
