# Supplementary material for: Admixture Mapping of African–American Women in the AMBER Consortium Identifies New Loci for Breast Cancer and Estrogen-Receptor Subtypes
Source: Front Genet. 2016 Sep 21;7:170. doi: 10.3389/fgene.2016.00170 (PMC5030764; doi:10.3389/fgene.2016.00170)
Supplement: TABLE S3 — Genomic regions with suggestive excess (negative Z-Score) and reduced (positive Z-score) African ancestry associated with breast cancer at 4 >|Z| ≤ 3.5 in case-only analyses and corresponding case-control results. [file Table_3.DOCX]

| Supplemental Table 3. Genomic regions with suggestive excess (negative *Z*-Score) and reduced (positive *Z*-score) African ancestry associated with breast cancer at 4 > \|*Z*\| ≥ 3.5 in case-only analyses and corresponding case-control results^a^ | | | | | | | | | |
| --- | --- | --- | --- | --- | --- | --- | --- | --- | --- |
| Locus | All breast cancers | | ER+ breast cancers | | ER- breast cancers | | TNBC | | Breast cancer GWAS SNPs |
|  | *Zc* / *Zcc* | OR^b^ (95% CI) | *Zc* / *Zcc* | OR^b^ (95% CI) | *Zc* / *Zcc* | OR^b^ (95% CI) | *Zc* / *Zcc* | OR^b^ (95% CI) |  |
| 2q24 | 1.7 / 1.5 | 0.92 (0.84-1.02) | -0.1 / -0.4 | 0.97 (0.87-1.10) | 3.6 / 2.6 | 0.81 (0.70-0.94) | 2.4 / 2.3 | 0.78 (0.64-0.95) | rs2016394 rs1550623 |
| 2q37 | -2.7 / -1.7 | 1.09 (0.99-1.21) | -1.2 / -1.1 | 1.07 (0.94-1.21) | -2.6 / -1.8 | 1.14 (0.97-1.34) | -3.8 / -3.1 | 1.41 (1.13-1.77) | -- |
| 3p24 | -3.7 / -1.7 | 1.09 (0.98-1.21) | -2.7 / -1.7 | 1.11 (0.98-1.26) | -2.3 / -1.6 | 1.13 (0.96-1.33) | -3.8 / -2.6 | 1.34 (1.08-1.67) | rs4973768 rs12493607 |
| 5p15 | -3.5 / -1.1 | 1.06 (0.95-1.18) | -0.8 / 0.7 | 0.95 (0.84-1.09) | -3.8 / -2.1 | 1.19 (1.00-1.42) | -2.8 / -1.6 | 1.20 (0.95-1.51) | rs10069690  rs2736100 |
| 8q24 | -2.7 / -1.6 | 1.09 (0.97-1.21) | -3.6 / -2.6 | 1.19 (1.04-1.36) | -0.1/ 0.4 | 0.96 (0.81-1.13) | 0.04 / 0.3 | 0.95 (0.76-1.18) | rs13281615 rs1562430 rs11780156 rs2392780 |
| 9q34 | -3.5 / -2.8 | 1.16 (1.04-1.28) | -2.8 / -2.2 | 1.15 (1.02-1.30) | -2.6 / -2.0 | 1.16 (0.99-1.37) | -1.8 / -1.7 | 1.19 (0.96-1.47) | -- |
| 18p11 | -1.1 / 1.5 | 0.92 (0.83-1.02) | 0.03 / 1.6 | 0.90 (0.79-1.02) | -1.5 / 1.0 | 0.98 (0.83-1.16) | -3.9 / -2.6 | 1.35 (1.07-1.71) | -- |
| 18q23 | -2.2 / -0.6 | 1.03 (0.93-1.14) | -0.1 / 0.8 | 0.95 (0.84-1.08) | -3.6 / -2.1 | 1.18 (1.00-1.39) | -2.8 / -1.8 | 1.21 (0.97-1.51) | -- |
| ^a^ 4 > \|*Z*\| ≥ 3.5 case-only analysis results are presented only for regions with case-control results \|*Z*\| ≥ 2.0, *P* < 0.05  ^b^ Odds ratio and 95% confidence interval per African allele estimated from the admixture mapping case-control analysis. Odd ratios were adjusted for individual African ancestry, study site, age, geographic region, and DNA source  TNBC = triple negative breast cancer  *Zc* = *Z*-score from the case-only analysis  *Zcc* = *Z*-score from the case-control analysis | | | | | | | | | |
