# Supplementary material for: Admixture Mapping of African–American Women in the AMBER Consortium Identifies New Loci for Breast Cancer and Estrogen-Receptor Subtypes
Source: Front Genet. 2016 Sep 21;7:170. doi: 10.3389/fgene.2016.00170 (PMC5030764; doi:10.3389/fgene.2016.00170)

Supplemental Figure 1. Plot of the top two principal components of genetic variation stratified by study in the AMBER consortium.

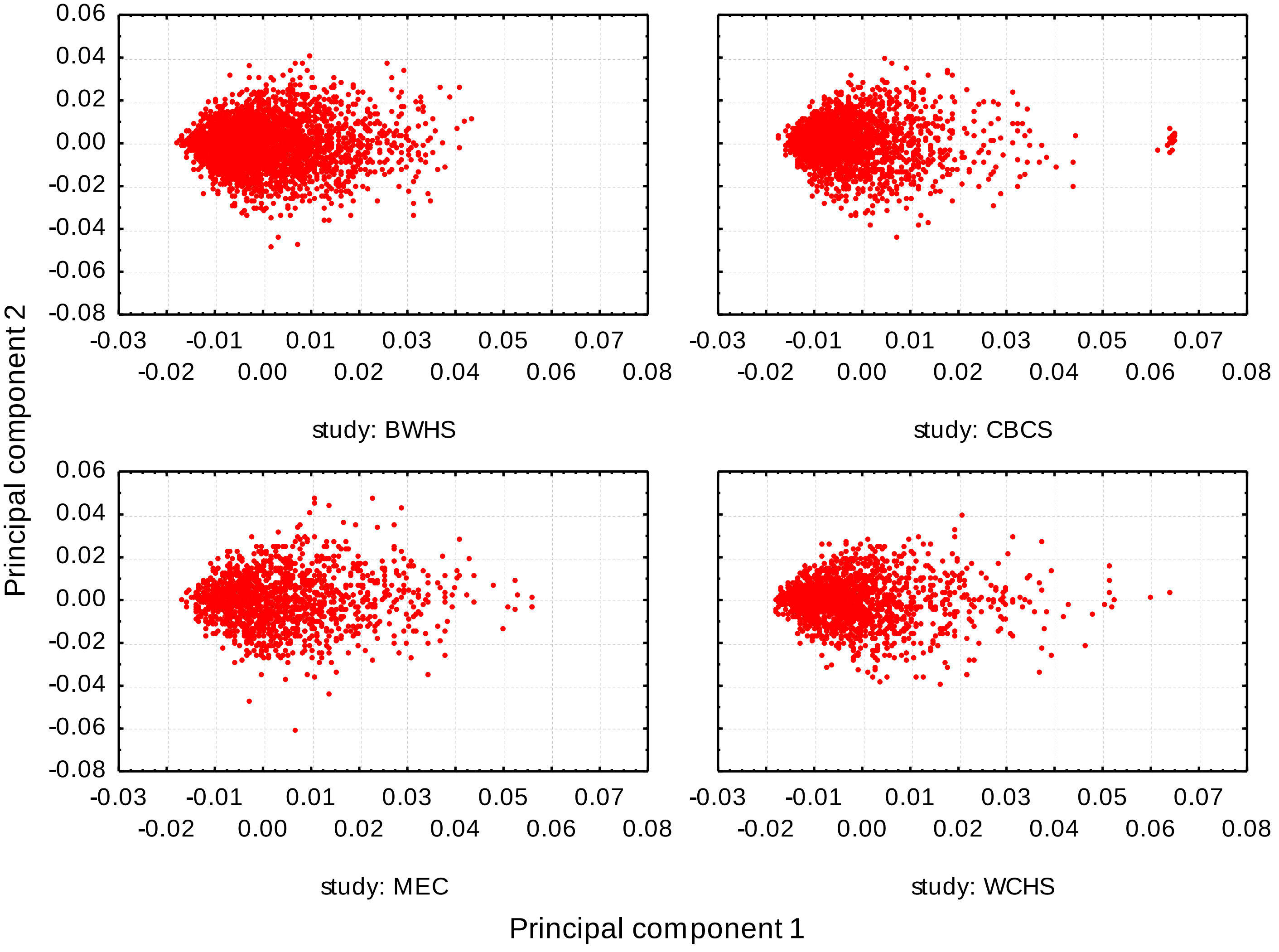

Supplement: Supplementary file 5 [file Image_1.PDF]
