# Supplementary material for: Admixture Mapping of African–American Women in the AMBER Consortium Identifies New Loci for Breast Cancer and Estrogen-Receptor Subtypes
Source: Front Genet. 2016 Sep 21;7:170. doi: 10.3389/fgene.2016.00170 (PMC5030764; doi:10.3389/fgene.2016.00170)

Supplemental Figure 2. Association plots of regions genome-wide significant regions identified through admixture mapping and with SNP associations at $P < 5.8 \times 10^{-6}$ (purple dots). Gray dots represent SNPs with no information about linkage disequilibrium.

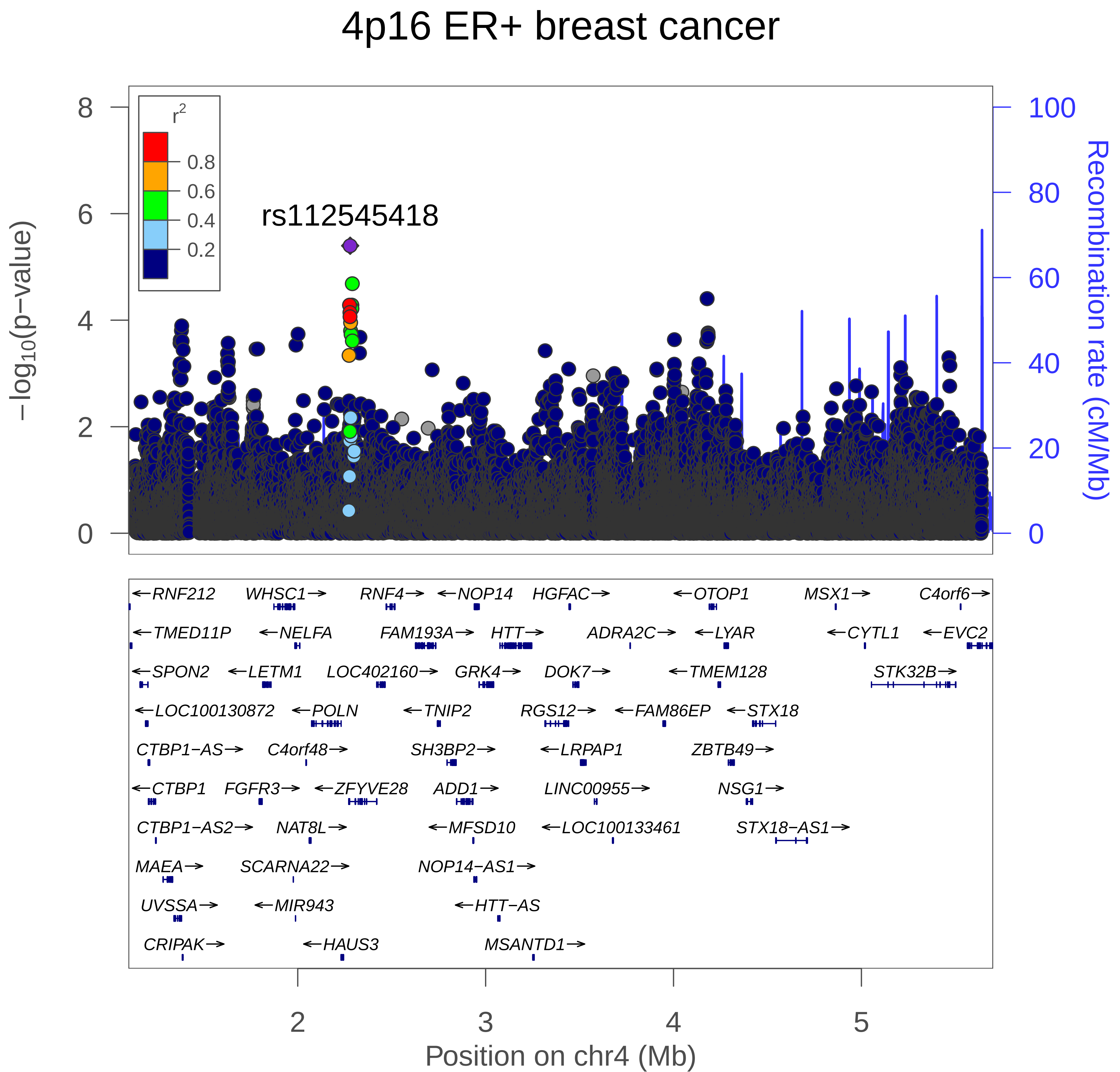
4p16 ER+ breast cancer
rs112545418
r2
0.8
0.6
0.4
0.2
−log10(p−value)
Recombination rate (cM/Mb)
8
6
4
2
0
100
80
60
40
20
0
←RNF212
WHSC1→
RNF4→
←NOP14
HGFAC→
←OTOP1
MSX1→
C4orf6→
←TMED11P
←NELFA
FAM193A→
HTT→
ADRA2C→
←LYAR
←CYTL1
←EVC2
←SPON2
←LETM1
LOC402160→
GRK4→
DOK7→
←TMEM128
STK32B→
←LOC100130872
←POLN
←TNIP2
RGS12→
←FAM86EP
←STX18
CTBP1−AS→
C4orf48→
SH3BP2→
←LRPAP1
ZBTB49→
←CTBP1
FGFR3→
←ZFYVE28
ADD1→
LINC00955→
NSG1→
CTBP1−AS2→
NAT8L→
←MFSD10
←LOC100133461
STX18−AS1→
MAEA→
SCARNA22→
NOP14−AS1→
UVSSA→
←MIR943
←HTT−AS
CRIPAK→
←HAUS3
MSANTD1→
2
3
4
5
Position on chr4 (Mb)

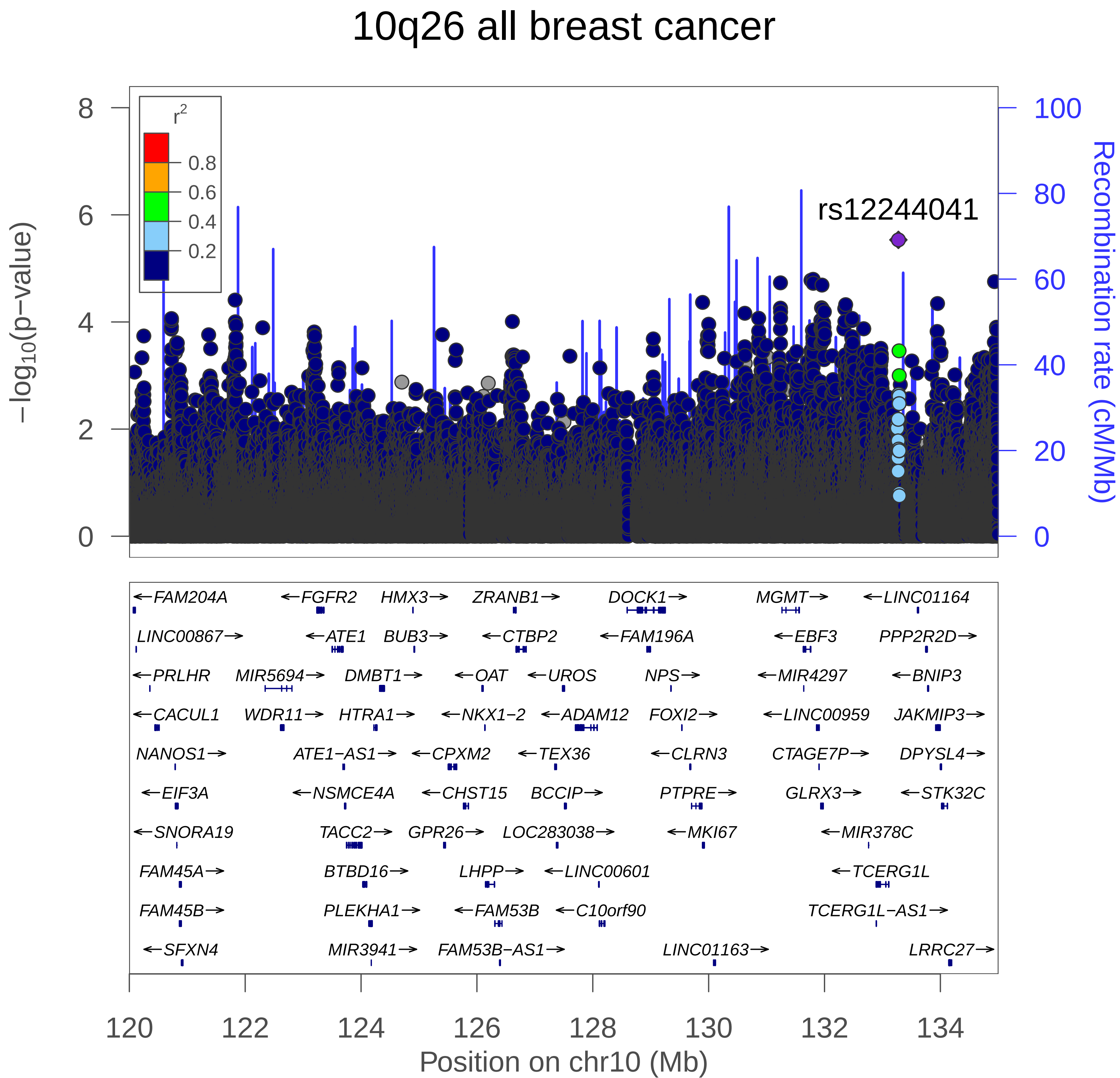

10q26 all breast cancer
r2
0.8
0.6
0.4
0.2
rs12244041
−log10(p−value)
Recombination rate (cM/Mb)
0
2
4
6
8
0
20
40
60
80
100
FAM204A
FGFR2
HMX3
ZRANB1
DOCK1
MGMT
LINC01164
LINC00867
ATE1
BUB3
CTBP2
FAM196A
EBF3
PPP2R2D
PRLHR
MIR5694
DMBT1
OAT
UROS
NPS
MIR4297
BNIP3
CACUL1
WDR11
HTRA1
NKX1−2
ADAM12
FOXI2
LINC00959
JAKMIP3
NANOS1
ATE1−AS1
CPXM2
TEX36
CLRN3
CTAGE7P
DPYSL4
EIF3A
NSMCE4A
CHST15
BCCIP
PTPRE
GLRX3
STK32C
SNORA19
TACC2
GPR26
LOC283038
MKI67
MIR378C
FAM45A
BTBD16
LHPP
LINC00601
TCERG1L
FAM45B
PLEKHA1
FAM53B
C10orf90
TCERG1L−AS1
SFXN4
MIR3941
FAM53B−AS1
LINC01163
LRRC27
120
122
124
126
128
130
132
134
Position on chr10 (Mb)

11q13 ER+ breast cancer

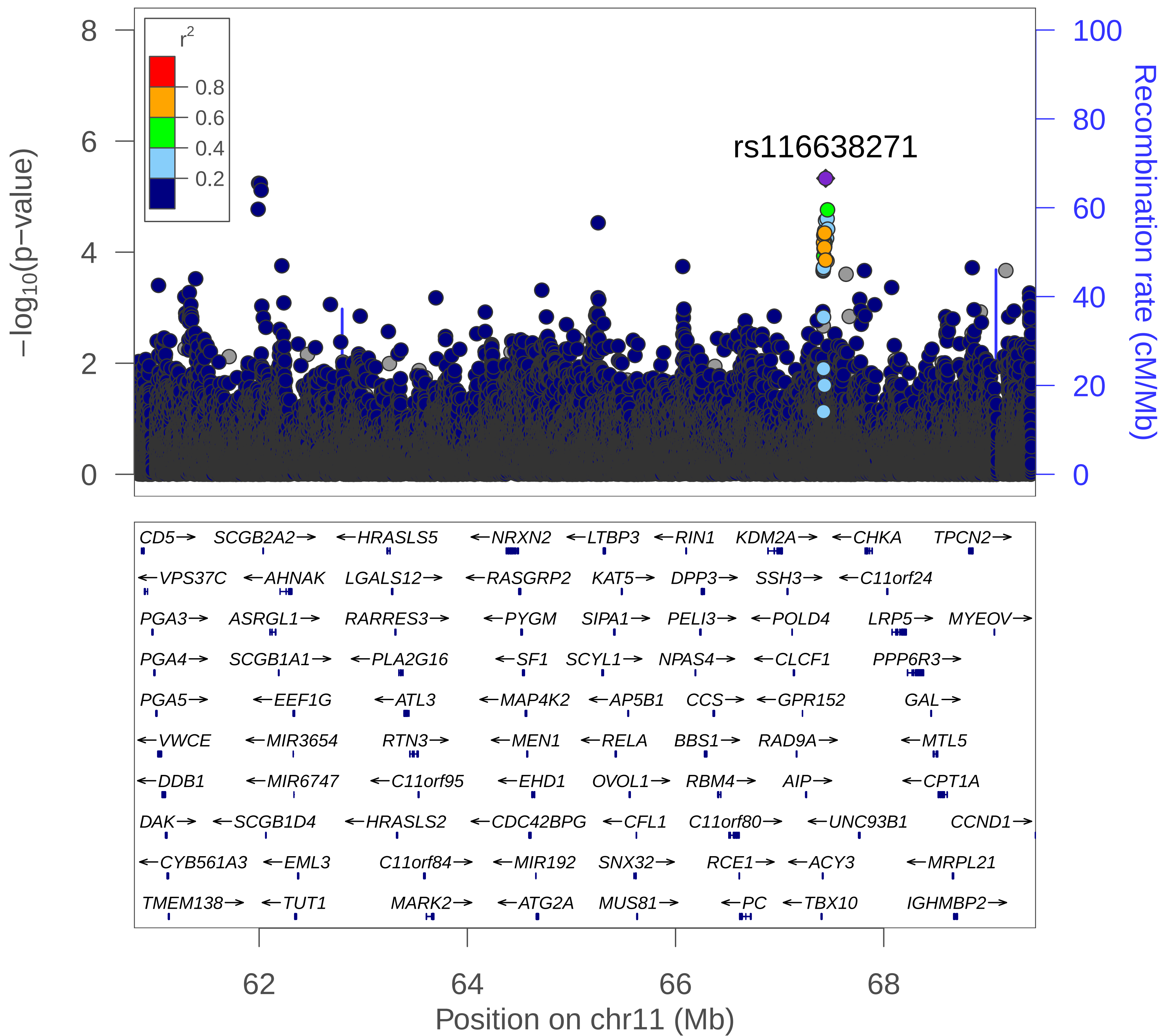

Supplement: Supplementary file 6 [file Image_2.PDF]
